# HLA Class II Alleles and Suicidal Behavior: Evidence from a Case–Control Study

**DOI:** 10.3390/ijms262010181

**Published:** 2025-10-20

**Authors:** Mihaela Elvira Cîmpianu, Mihaela Laura Vică Matei, Ștefana Bâlici, Gheorghe Zsolt Nicula, Elena Maria Domșa, Teodora Cîmpianu, Sergiu Ionica Rusu, Horia George Coman, Costel Vasile Siserman

**Affiliations:** 1 Department of Cellular and Molecular Biology, Iuliu Haṭieganu University of Medicine and Pharmacy, 400006 Cluj-Napoca, Romania; mihaela.cimpianu@uab.ro (M.E.C.); mvica@umfcluj.ro (M.L.V.M.); sbalici@umfcluj.ro (Ș.B.); gnicula@umfcluj.ro (G.Z.N.); 2 Department of Social Sciences, “1st December 1918” University of Alba Iulia, 510009 Alba Iulia, Romania; 3 Legal Medicine Institute, 400006 Cluj-Napoca, Romania; cvsiserman@gmail.com; 4 Faculty of Medicine, Iuliu Haṭieganu University of Medicine and Pharmacy, 400006 Cluj-Napoca, Romania; cimpianu.teodora@elearn.umfcluj.ro; 5 Faculty of Sociology and Social Work, Babeș-Bolyai University, 400084 Cluj-Napoca, Romania; sergiu.rusu.ibc@gmail.com; 6 Romanian Institute for Evaluation and Strategy-IRES, 400495 Cluj-Napoca, Romania; 7 Department of Medical Psychology, Iuliu Haṭieganu University of Medicine and Pharmacy, 400006 Cluj-Napoca, Romania; hcoman@umfcluj.ro; 8 Department of Legal Medicine, Iuliu Haṭieganu University of Medicine and Pharmacy, 400006 Cluj-Napoca, Romania

**Keywords:** suicide attempts, suicide behavior, HLA, genotype, haplotype, SSP-PCR, psychosocial determinants

## Abstract

Suicidality is a complex multifactorial phenomenon strongly associated with major depression and other psychiatric disorders. Building on evidence implicating the Major Histocompatibility Complex (MHC) in modulating the immune and inflammatory processes characterizing psychiatric disorders, we hypothesized that specific *HLA-DQB1* and *HLA-DRB1* variants may contribute to an increased genetic susceptibility to suicidal behavior. Human Leucocyte Antigen (HLA) typing by sequence-specific primers (PCR-SSP) was performed on a sample of 196 individuals, including 70 non-lethal suicide attempters, 28 cases of completed suicide, and matched controls. The *HLA-DQB1 02/06 (RR 1.60, CI95% 1.22–2.09, *p* = 0.03 *) and *HLA-DRB1 11/15 (RR 1.70, CI95% 1.3–2.24, *p* = 0.04 *) genotypes and the *HLA-DRB115~DQB103* haplotype (RR 1.58, CI95% 1.22–2.04, *p* = 0.03 *) were found to favor suicidal behavior. Psychosocial determinants associated with an increased suicidal risk were bereavement of close relatives (linked with HLA-DQB1*02), memory dysfunction (HLA-DQB1*06), disillusionment (HLA-DRB1*07 and HLA-DRB1*15), and self-harm (HLA-DRB1*15). Our findings support the contributory role of HLA polymorphisms in shaping susceptibility to suicidal behavior.

## 1. Introduction

Claiming over 720,000 annual deaths, suicide is a serious multi-factorial public health problem affecting mainly elders [1,2] and youngsters [3,4,5,6], or vulnerable groups who experience discrimination [7,8]. Mental disorders (major depressive disorder in particular), often in conjecture with alcohol or drug abuse, are important indicators of high suicide risk [9,10,11,12,13,14], with most suicide attempters being documented to suffer from psychiatric disorders [15]. Those with a history of suicide attempts are the most likely to repeat such action, particularly over the first year since their previous attempt [11,16,17,18]. Men commit more suicides as they tend to use violent methods, while women have significant more suicide attempts, primarily involving drug abuse [9].

The presence of a mental disorder alone does not necessarily lead to suicide, unless specific symptoms and other clinical or demographic factors increase the probability of self-harm in subjects affected by psychiatric conditions [10,11]. According to the stress-diathesis model of suicide, proximal risk factors (e.g., mental illness, physical illness, psychosocial crises, substance use, availability of lethal means) act as triggers when interacting with distal risk factors (genetic characteristics, personality features, childhood trauma, neurobiological disturbances) under stressful circumstances [19].

Several epidemiological studies suggested that, while mental disorders can predict the onset of suicide ideation, their power to forecast suicide plans or attempts may be weaker [20,21]. In contrast, certain medical conditions (e.g., a diagnosis of cancer or multiple physical illnesses) could double the suicide risk [22].

As the risk of suicide increases with every comorbidity [9,22], the effects of comorbidities need to be taken into consideration in a more rigorous manner [10]. While medical (e.g., chronic pain) or socio-economical (e.g., financial or relationship problems) factors may account for impulsive violent decisions, several other medical, psychological and environmental factors contribute to planned self-harm actions. Several theories attempted to explain this complex phenomenon [23,24,25,26].

The influence of certain psychological and environmental determinants was the object of numerous studies. A history of violence, abuse or other traumatic experiences, a perceived sense of isolation, were strongly associated with suicidal behavior [7]. Certain personality traits (impulsivity, anger, aggression, neuroticism) were found to increase the risk of suicide behavior [27], impulsiveness being seen as a consistent risk factor in the progression from ideation to suicidal behavior [16]. Highlighting the complex interplay observed in suicidal behavioral processes, a meta-analysis on suicide risks found that 50% of patients considered to be at low risk actually do commit suicide, while 95% of the high-risk patients do not [28].

Studies on twins and adoption cases have outlined the existence of a genetic vulnerability for suicidal behavior, independent of markers presumed to influence various mental illnesses significantly associated with suicidal behavior [29]. Researchers found that first degree relatives of people who commit suicide (including dizygotic twins) present a double risk to commit suicide compared to the general population, while for monozygotic twins the relative risk increases approximately 11-fold. Structural analysis of published data suggests a heritability for complete suicide of about 43% [30].

The heritability hypothesis has been supported by studies on immigrants, confirming similarities in suicide rates between these and their country of origin populations [31].

Suicidal behavior was associated with the expression of various candidate genes or polygenic systems (the serotonergic system, the dopaminergic system, the hypothalamic–pituitary–adrenal axis, the brain derived neurotrophic factor, etc.). Higher suicidal risks were documented when abnormalities in the serotonergic system, the hypothalamic–pituitary–adrenal (HPA) axis, lipid metabolism, immunity or neuroplasticity were observed [16,29,32,33,34].

Objective indicators of biological processes, the biomarkers can be quantified by observing changes in gene expression or in the structure of various proteins, or by tracking chemical changes in metabolites detectable in the central and peripheral nervous systems. They can facilitate medical diagnosis of medical conditions and can provide indications regarding the effectiveness of various treatments and suggesting new therapeutic targets [35,36,37]. Since the detection in 1958 of the first Human Leucocyte Antigen (HLA), later classified as HLA A2, the Major Histocompatibility Complex (MHC) region on chromosome 6 was intensively researched due to the highly polymorphic character of its hundreds of genes and their major impact in histocompatibility [38,39] and immunology [40,41,42]. Their high allelic diversity and low recombination rates have made them invaluable in transplant matching [38]. Acting as receptors for infectious agents involved in psychiatric pathologies, HLA are notable for their ability to confer susceptibility or resistance to various immune-mediated disorders [40,43]. Their primary function is to present endogenous antigens to CD8+ T cells (HLA class I) or exogenous antigens to CD4+ T cells (HLA class II molecules), thus initiating adaptive immune responses [44]. However, HLA genes were associated with various other medical conditions, including psychiatric diseases [45,46,47,48,49], multiple sclerosis [42,50,51], epilepsy [52], acquired immunodeficiency syndrome (AIDS), cervical cancer, Hodgkin’s disease [50], ankylosing spondylitis [51] etc.

HLA variation may contribute to suicidal behavior through immune–brain pathways that converge on microglial activation and neurotransmission. preliminary case–control studies have reported allele-level signals, including increased risk with HLA-DQB1*02 in suicide attempters and decedents [53,54]. Mechanistically, class II HLA influences antigen presentation and cytokine tone, which can trigger microglial activation. Postmortem work showed differences in HLA-DR–positive (MHC-II) microglia in suicidal decedents—including in the dorsal raphe nucleus, a key serotonergic hub—while recent narrative and systematic reviews synthesize convergent HLA-DR/microglial changes across anterior cingulate, prefrontal cortex, hippocampus, and thalamus, with regional heterogeneity and diagnostic moderators [55,56,57].

Genome-wide association studies (GWAS) rejuvenated interest in the involvement of the immune system in psychiatric conditions (e.g., schizophrenia, bipolar disorder, autism), of particular interest being the assessment of HLA gene variants’ risk-enhancing or protective potential regarding subgroups of psychiatric disorders [58]. New susceptibility loci identified by the application of polygenic risk scores in GWAS may highlight biological pathways partially shared by suicidality and certain psychiatric disorders [59].

Suicide is preventable, provided timely evidence-based interventions are made available through comprehensive prevention programs [7]. Blocking access to suicide means such as firearms or frequently used places (bridges, railways), or to potentially poisonous medication, is the basis of any prevention measures. The internet may provide a multitude of information for help-seekers, but professionals’ training in primary and secondary care remains vital [9,60].

Despite decades of research, methods to consistently distinguish high-risk from low-risk patients are lacking. Risk categorization is rather useless if there are no rational interventions that should be provided to high-risk patients (most of whom will not commit suicide), but not to low-risk patients that actually provide about half of all suicide cases [28]. National-wide prevention strategies are needed in this pursuit [7,61,62].

The identification of specific biological markers associated with self-harm may help implementing prevention strategies and developing efficient therapeutic schemes [16]. Considering the MHC implication in modulating the immune or inflammatory processes that characterize psychiatric disorders, we hypothesized that specific alleles, genotypes and haplotypes within the *HLA-DQB1* and *HLA-DRB1* loci are significantly associated with suicidal behavior, the investigation of these markers leading to the identification of new correlations between HLA polymorphisms and genetic susceptibility to suicidal behavior.

## 2. Results

In line with this objective, the association of *HLA class II* alleles with suicidal behavior was analyzed on a case group of 98 (67 men and 31 women, mean age 39.69 ± 1.79 years), of which 74 from urban areas. The case group included 70 subjects with documented non-fatal suicidal behavior and 28 individuals with completed suicide. The control cohort included 98 participants (67 men and 31 women; mean age 40.88 ± 1.39 years), 57 of whom were from rural areas, all without any history of suicidal behavior. The following subsections present the distribution of alleles, genotypes, and haplotypes for each locus, together with their observed relationships to psychosocial determinants.

### 2.1. HLA-DQB1 and DRB1 Alleles Genetic Associations with Suicidal Behavior

Results are presented separately for each locus in the subsections below.

#### 2.1.1. Genetic Associations of *HLA-DQB1* Alleles and Genotypes with Suicidal Behavior

Cross-tabulation between the dependent variable (suicide attempt) and the independent variables, *HLA-DQB1* allele frequencies, did not produce statistically significant results (as shown in Table 1). Similar data regarding the *HLA-DQB1* genotypes are shown in Table 2. Statistical significance was observed for two genotypes: HLA-DQB1*02/*05 was more frequent in controls than in cases (protective role, relative risk RR = 0.61) and HLA-DQB1*02/*06 occurred more often among cases than controls (susceptibility role, relative risk RR = 1.60, odds ratio OR = 3.69).

#### 2.1.2. Genetic Associations of *HLA-DRB1* Alleles and Genotypes with Suicidal Behavior

Similar analyses were made for the *HLA-DRB1* alleles (highlighted in Table 3) and genotypes (detailed in Table S1) likely to influence suicidal behaviors in the analyzed sample. Significant results were observed for the HLA-DRB1*04 allele (protective role, RR = 0.61, OR = 0.43, CI 0.20–0.90, *p* = 0.03) and the DRB1*01/*03 genotype was observed with a significantly higher frequency in the control cohort compared to the case cohort (RR = 0.33, OR = 0.19, CI 0.04–0.89, *p* = 0.04), whereas the HLA-DRB1 *11/*15 genotype occurred more frequently among cases than controls (RR = 1.70, OR = 5.22, CI 1.13–24.12, *p* = 0.04).

### 2.2. Distribution of HLA-DRB1~DQB1 Haplotypes and Their Association with Suicidal Behavior

Out of the 196 possible *HLA-DRB1*~*DQB1* haplotypes, 54 were identified in the analyzed groups, two of which presenting statistical significance: DRB1*04~DQB1*03 was enriched among controls relative to cases (protective role, RR = 0.62, OR = 0.45, CI 0.23–0.86, *p* = 0.02), and DRB1*15~DQB1*03 was overrepresented in cases (susceptibility role, RR = 1.58, OR = 3.59, CI 1.17–11.01, *p* = 0.03). Results are illustrated in Table S2.

### 2.3. Correlations of Suicidal Behaviors with Psychosocial Determinants in HLA-DQB1 and DRB1 Alleles

The analysis of psychosocial determinants in relation to HLA allelic distribution was carried out using data collected through the sociological questionnaire and medical records. Demographic and socio-economic variables, psychiatric diagnoses, quality of life, suicide history, and socio-emotional loneliness were considered. Table 4, Table 5 and Table 6 summarize the correlations observed between suicidal behaviors and these psychosocial determinants in the case group, for the *HLA-DQB1* and *HLA-DRB1* alleles, respectively.

#### 2.3.1. Correlations of *HLA-DQB1* Alleles with Psychosocial Determinants of Suicidal Behavior

HLA typing of the *DQB1* locus was performed using the SSP-PCR method, allowing the identification of allelic distributions within the study cohort. Based on these results, correlations between *HLA-DQB1* alleles and psychosocial determinants of suicidal behavior were subsequently examined.

#### 2.3.2. Correlations of *HLA-DRB1* Alleles with Psychosocial Determinants of Suicidal Behavior

For the *HLA-DRB1* locus, molecular typing allowed the identification of specific allelic profiles, which were further examined for correlations with psychosocial determinants of suicidal behavior.

The study highlighted significant correlations with several psychosocial determinants of suicidal behaviors. Susceptibility factors proved to be the death of close relatives (for those presenting the DQB1*02 allele), memory disorders (in DQB1*06 subjects), disillusionment (for those exhibiting the DRB1*07 or DRB1*15 alleles), and self-aggression (in patients presenting the DRB1*15 allele). The absence of family or social problems in case subjects exhibiting the DRB1*01 allele and of drug addiction in those presenting the DQB1*03 allele acted as protective factors.

## 3. Discussion

The neurobiology of suicide remains a hot research topic. Biomarkers with promising clinical potential include indices of serotonergic function, inflammation, lipid metabolism or neuronal plasticity. The serotonergic system predisposes suicidal behavior, higher serotonin-1A autoreceptor binding being associated with more severe suicidal ideation and lethal attempts. Studies of HPA axis dysfunction found that suicide attempters exhibiting high aggression and impulsivity had the most marked cortisol response. An increase in metabotropic glutamate receptor type 5 was observed in suicidal individuals with posttraumatic stress disorder. And the list grows day after day [19,63].

Aberrations in inflammatory cytokines have been reported in several neuropsychiatric conditions, including MDD, schizophrenia and bipolar disorders. It is documented that inflammation can trigger depressive symptoms and is associated with suicidality based on studies involving patients who receive interferon (IFN)-based or interleukin-2(IL-2) immunotherapy [64].

Despite significant progress in recent decades, a poor understanding of the neurobiological bases of suicide due to difficulties in accessing the human brain complexity translates in the absence of effective prediction models. However, genome-wide analysis have contributed to improving our understanding of suicidal behavior [27,28,32,65]. A huge benefit is that GWAS are not based on prior assumptions, a simple regression analysis being used to systematically test each biallelic SNP across the genome for association with a trait or disease [66]. Recent studies have consistently pointed out a shared genetic architecture of suicidal behavior with psychiatric disorders or various other determinants [65,67,68].

For decades the HLA locus has been perceived as a susceptibility locus for psychiatric disorders, conditions frequently associated with suicidal behavior. The HLA system also plays a prominent role in regulating immuno-inflammatory processes in viral infections, which proved to be important during the COVID-19 pandemic [58,69,70]. *HLA* polymorphisms have a significant impact on a long list of diseases. From an evolutionary perspective it has been speculated that, during the evolution of species, certain *HLA class I* alleles less prone to mutations (e.g., HLA-B*27, HLA-B*51, HLA-B*57:01 and HLA-C*06, the ones exhibiting the strongest protective effect against HIV infections) have been selected in the course of evolution by devastating epidemics as their immunodominant peptides generated stronger immune responses. While class I are generally linked to inflammatory disorders, *class II HLAs* are frequently associated with autoimmune diseases [71].

HLA genes are the strongest risk factors for most autoimmune diseases, and some mechanisms have now been elucidated. In the celiac disease gluten peptides modified by the transglutaminase are loaded into the groove of specific DQ2 molecules, triggering a TCR-mediated cytokine cascade. In the chronic beryllium disease, the binding of beryllium to the HLA-DP molecules (facilitated by the presence of Glu69 and a negatively charged amino acid at P4 of the peptide and two other negatively charged amino acids in the groove) triggers a beryllium-specific polyclonal T-cell response leading to inflammation and tissue damage. Based on these and similar findings, it was proposed that the association between *HLA-class I* and *HLA-class II* with diseases is based on similar mechanisms in which either a specific antigen activates a strong T cell response, or interference of certain molecules with the peptide repertoire generates an inflammatory cascade [71].

Another theory involves the gut–brain axis linking emotional and cognitive centers of the brain with peripheral intestinal functions under the influence of gut microbiota. The bidirectional interaction between microbiota and the gut–brain axis involves signaling from gut-microbiota to brain and from brain to gut-microbiota by means of neural, endocrine, immune, and humoral links [72].

It was found that HLA alleles correlate with oral microbiota differences linked to suicidal ideation, while altered gut communities were observed in psychiatric disorders [73].

The impact of the COVID-19 pandemic on suicide rates has been reflected in several other studies associating it with significant levels of distress, anxiety, fear of contagion and depression [74,75,76]. Consistent with expectations shaped by earlier outbreaks (Severe Acute Respiratory Syndrome, Ebola), projections at the outset were pessimistic. Economic uncertainty or depressive states induced by social isolation imposed during the COVID-19 pandemic were estimated to produce a significant impact on the suicide phenomenon [75,76,77]. However, a meta-analysis that assessed across various populations from 33 countries changes in the prevalence of suicidal ideation, suicide attempt, and suicide mortality rate before and during the COVID-19 pandemic (as of December 2022) found that suicide death rates did not change significantly, but suicidal ideation and suicide attempt were more prevalent compared with the pre-pandemic period [78].

Although a GWAS on populations of European ancestry found no direct associations between HLA and an increased risk of depression, 14 HLA alleles (including HLA-DQB1*02, *03, HLA-DRB1*03, *07, *13, *15) have been shown to be associated with several autoimmune diseases (multiple sclerosis, Crohn’s disease, primary adrenocortical insufficiency, systemic lupus erythematosus, psoriasis vulgaris, type 1 diabetes mellitus). The MHC region was linked to schizophrenia (frequently associated with suicide, along with major depression and bipolar disorder), highlighting the differential functions (protective versus risk factors) attributable to HLA molecules. Strong evidence for a bidirectional relationship with major depression, the main risk factor for suicidal behavior, was observed for HLA-DQB1*02 and HLA-DRB1*03 [79]. A recent GWAS demonstrated that the genetic diversity of *HLA-DRB1* and *HLA-DQB1* alleles modulates responses to treatment with lithium-containing drugs in bipolar affective disorders [80]. Another study has found that *HLA-DR* polymorphisms were involved in the SARS-CoV-2 infection, suggesting susceptibility roles for HLA-DRB1*13 and HLA-DRB1*15 [81]. The latter was also reported as the highest risk allele to be associated with multiple sclerosis [82].

A Tunisian study found that the HLA-DQB1*02 allele is the most susceptible, and HLA-DQB1*05 the most protective, in/against the development of schizophrenia [47]. On the other hand, an American study on a large cohort of deceased organ donors, mainly Caucasians, which associated intentional violent death with 21 *HLA-DR* and 10 *HLA-DQ* alleles, found that both HLA-DQB1*02 and HLA-DQB1*05 were significantly associated with increased risks of violent death [50].

Exploring the interface between commensal microbes residing in the oral cavity and the MHC, an American study on university students with suicidal ideation has found that HLA Class II DQA1*01, DRB1*15, DRB3*02, DPB1*01/*05 and HLA Class I C*08 and A*30 were positively associated with the incidence of suicidal ideation, while DRB1*04, DQA1*03, DQB1*03, and DRB3*01 appeared to be protective [83].

Our study complements the existing data attempting to explain the facets of this serious health problem. We found that the HLA-DQB1*02 allele appeared in two genotypes: HLA-DQB1*02/*05, posing a protective role (RR 0.61, *p* = 0.02), and HLA-DQB1*02/*06, susceptible to favor suicidal behavior (RR 1.60, OR 3.69, *p* = 0.03). This study identified that the HLA-DRB1*04 allele (RR 0.6, *p* = 0.03) and the HLA-DRB1*01/*03 genotype (RR 0.33, *p* = 0.04) act as protective factors against developing suicidal behavior, in contrast to the HLA-DRB1*11/*15 genotype (RR 1.70, OR 5.22, *p* = 0.04). Other susceptibility genotypes absent in the control group were HLA-DRB1*07/*13 (RR 2.05), DRB1*13/*13 (RR 2.03), and DRB1*16/*16 (RR 2.04). In another study [84] the HLA-DRB1*03 allele, a component of the HLA-DRB1*01/*03 genotype, was identified as exerting a protective role against schizophrenia. We found two haplotypes involving the HLA-DQB1*03 allele that presented statistical significance: HLA-DRB1*04~DQB1*03 (protective role, RR = 0.62, OR = 0.45, *p* = 0.01), and HLA-DRB1*15~DQB1*03 (susceptibility role, RR = 1.58, OR = 3.59, *p* = 0.03), respectively.

Compared to a study published by us in 2023 [54], the current study confirms as a possible susceptibility factor to suicidal behavior only the HLA-DQB1*02/*06 genotype, and as a protective factor the HLA-DQB1*02/*05 genotype. The previous study additionally showed positive correlations with suicidal behavior for the HLA-DQB1*02, *03, and *06 alleles, the HLA-DQB1*02/*03, -DRB1*12/*15, and -DRB1*07/*13 genotypes, as well as the HLA-DRB1*07~DQB1*06 and HLA-DRB1*13~DQB1*02 haplotypes, respectively, while the HLA-DQB1*04 allele, the HLA-DQB1*03/*05 genotype, and the HLA-DRB1*13~DQB1*03 haplotype are negatively correlated [54]. In contrast to that study, we now found positive correlations with suicidal behavior for the HLA-DRB1*11/*15 genotype and the HLA-DRB1*15~DQB1*03 haplotype, respectively, and negative correlations for the HLA-DRB1*04 allele, the HLA-DRB1*01/*03 genotype, and the HLA-DRB1*04~DQB1*03 haplotype. A Romanian study from 2019 [53] presents different results, with the HLA-DQB1*02 allele and the HLA-DQB1*02/*03 genotype being positively correlated, and the HLA-DQB1*05 allele negatively correlated with suicidal behavior.

Statistical analysis revealed various correlations between alleles occurring in the case group and a series of psychosocial determinants of suicidal behavior. When analyzing the *HLA-DQB1* alleles (Table 4) for possible links between drug addiction and suicide, a significant protective influence (*p* = 0.037) was found for the HLA-DQB1*03 allele (a frequency of 76.8% among addiction-free individuals). The death of close relatives proved to be a susceptibility factor for carriers of the HLA-DQB1*02 allele (frequency 70.6%, *p* = 0.046), a similar role exerting memory dysfunctions for subjects presenting the HLA-DQB1*06 allele (carrier frequency 83.3%, *p* = 0.033). By altering inhibitory processes, memory contributes to the suicidal risk by preventing individuals from using past experiences to solve current problems [85]. The analysis of *HLA-DRB1* correlations with psychosocial determinants (Table 5 and Table 6) highlighted the protective influence of a favorable family and social environment in HLA-DRB1*01 allele carriers. Disillusionment was identified as a susceptibility factor for the development of suicidal behavior among patients with the HLA-DRB1*07 alleles (79.2%, *p* = 0.043) and HLA-DRB1*15 (85.2%, *p* = 0.004), as was self-aggression in HLA-DRB1*15 allele carriers (66.7%, *p* = 0.01).

Our study was limited to a predominantly Caucasian population with reduced ethnic variability, from a relatively small area: the control group pooled six Transylvanian counties subordinated to the Cluj-Napoca Institute of Legal Medicine (ILM), while the case group included patients admitted to the Psychiatry Clinic within the Cluj County Clinical Hospital, and deceased persons autopsied at the ILM. The absence of a comprehensive database of HLA allele frequencies concerning Romanian citizens who commit suicide, covering the main ethnic subgroups, negatively influenced the study. Acknowledgeable limitations of this study include the small sample sizes encountered in several sub-group analyses. These limited cell counts inherently result in reduced statistical power and, in some instances, undefined odds ratios (ORs). Consequently, findings derived from these specific comparisons should be interpreted with caution and are primarily considered exploratory or hypothesis-generating, rather than definitive. Given the exploratory nature of this study, the Bonferroni test to adjust for multiple comparisons was not applied. Instead, we focused on interpreting the results in light of their biological relevance and consistency.

Ultimately, generating a clearer, nationwide picture of the phenomenon requires further studies employing larger, representative nationwide samples and advanced modeling algorithms specifically designed for a more robust and detailed assessment of suicide risks [86,87,88,89,90]. The identification of several HLA alleles and haplotypes potentially associated with both increased susceptibility (e.g., HLA-DQB1*02/*06, HLA-DRB1*15) and protection (e.g., HLA-DRB1*04, HLA-DQB1*03) suggests several promising directions for future research. Integrating genetic information with clinical and psychosocial risk factors could refine predictive models. Individuals presenting various risk factors who also carry high-risk HLA alleles could be recommended for more intensive clinical monitoring and early intervention. Our results suggest that HLA genotyping could serve as a valuable biomarker to identify vulnerable individuals in high-risk populations. If replicated in larger cohorts, these findings could guide personalized treatment.

## 4. Materials and Methods

### 4.1. Study Population Structure and Institutional Sources

A total of 196 participants were included in this case–control study, divided into two equal groups of 98 individuals each. The selection process and data acquisition followed a structured, ethically approved protocol, based on institutional collaboration with two key medical entities in Cluj-Napoca, Romania.

#### 4.1.1. Case Group Constitution

The case group consisted of individuals identified through two institutional sources:

- Psychiatric Clinic of the Cluj County Emergency Hospital:

A cohort of patients admitted for psychiatric care with at least one documented non-lethal suicide attempt. These cases were classified under International Classification of Diseases—10 (ICD-10) diagnostic codes:

○ X6x—Intentional self-poisoning
○ X7x—Intentional self-harm

All patients were evaluated and confirmed by clinical psychiatrists. Selection was based on medical records and diagnostic coding at admission. Clinical information was supplemented with informed consent and questionnaire-based data collection.

- Institute of Legal Medicine, Cluj-Napoca:

The second subgroup of the case group comprised individuals who had died by suicide. These cases were officially recorded as completed suicides in the medico-legal documentation of the institute and confirmed through forensic autopsy reports and toxicological analyses.

All individuals included in the case group were over 18 years of age at the time of sample acquisition. Cases with incomplete diagnostic information or suspected sample degradation were excluded.

#### 4.1.2. Control Group Constitution

The control group was formed from 98 unrelated individuals who underwent routine DNA paternity testing at the Molecular Biology Laboratory of the ILM in Cluj-Napoca. No familial relationships were identified within the control group or between case–control participants.

### 4.2. Data Acquisition Protocols and Preanalytical Handling

#### 4.2.1. Clinical and Demographic Data Collection

For living subjects (patients and controls), data were collected via a standardized sociological questionnaire, administered by trained research staff. The instrument included:

- Demographic information (age, sex, area of residence)
- Socioeconomic indicators
- Psychiatric diagnostic history (ICD-10 and ICD-10-CM coding)

Data from deceased individuals were obtained retrospectively from official medico-legal records, which included:

- Autopsy reports
- Toxicological screenings

#### 4.2.2. Sample Collection and Transport

- For living participants: Peripheral venous blood samples were collected in Ethylenediaminetetraacetic acid (EDTA)-coated vacutainer tubes by qualified personnel under sterile conditions. Samples were transported on ice.
- For deceased individuals: Biological specimens were collected during autopsy and preserved following standard medico-legal forensic protocols.

### 4.3. DNA Isolation and Quality Control Protocol

Peripheral blood was collected in EDTA tubes and processed using the Ready DNA Spin Kit (inno-train Diagnostik GmbH, Kronberg, Germany). The procedure relies on DNA binding to silica columns in the presence of chaotropic salts and involves the following steps:

- Cell lysis with Buffer 1, Proteinase K, and RNase
- Ethanol precipitation
- Column-based purification with Buffer X and Buffer 2
- Elution in 200 µL TRIS buffer

The resulting DNA was quantified and assessed for purity using the Pearl^®^ Implen nanophotometer (Implen GmbH, Munich, Germany), targeting a 260/280 absorbance ratio of 1.8 ± 10%. The expected DNA yield per sample was 5–10 µg.

### 4.4. HLA Genotyping: PCR-Based Allele Detection Technologies

Two molecular approaches were employed for HLA class II allele detection:

#### 4.4.1. Fluorescence-Guided Typing with HLA-FluoGene *DRDQ*

A high-throughput typing strategy was implemented using the HLA-FluoGene DRDQ kit (inno-train Diagnostik GmbH, Kronberg, Germany). This system utilizes sequence-specific primers (SSP-PCR) and fluorescence-labeled oligonucleotide probes for target-specific amplification and internal control detection (*HGH* gene).

Key operational details:

- DNA diluted to 1 ng/μL (±50%)
- PCR amplification on a G-Storm thermocycler (LabTech International, Rotherham, UK)
- Fluorescence signals detected using FluoVista Analyzer (endpoint) or FluoQube (real-time)
- Data processed with FluoGene Software(V. 1.8.0) evaluating Q-values and CT thresholds

PCR conditions (preset protocol):

- Initial denaturation: 96 °C for 2 min
- 38 cycles: 95 °C for 15 s, 60 °C for 49 s (fluorescence read)
- Ramp rate: 2.5 °C/s
- Detection channels: Blue, Green, Orange

#### 4.4.2. Gel-Based Typing with HLA-Ready Gene Kits

A complementary method was used on a subset of samples, employing HLA-Ready Gene DR and DQ kits( inno-train Diagnostic GmbH, Kronberg, Germany ) This system uses dried, pre-aliquoted primer mixes and gel electrophoresis for result visualization.

Workflow features:

- PCR amplification via ReadyGene protocols
- Electrophoretic separation on agarose gel
- Band pattern interpretation using Ready Gene Online browser-based software (V. 1.2.0.0)
- Integrated positive/negative controls and virtual allele mapping interface

### 4.5. Data Structuring, Statistical Analysis and Statistical Environment

Data tables were generated using Microsoft Excel from the Microsoft Office 2019 software group (Microsoft Corp., Redmond, WA, USA), while the Epi Info™ software (version 7.2.4.0) developed by the Centers for Disease Control and Prevention (Atlanta, GA, USA) and R v4.3.x (R Foundation for Statistical Computing, Vienna, Austria) were used for statistical analysis. Statistical significance was set at α = 0.05 (two-tailed). Descriptive statistics were expressed as counts and percentages for categorical variables and as mean (±SD) or median [IQR] for continuous variables, as appropriate. Group comparisons for categorical data (case vs. control; allele/genotype/haplotype frequencies; psychosocial determinants) were performed using Pearson’s chi-square test with Yates’ continuity correction where applicable. When chi-square assumptions were violated (expected cell counts < 5 in >20% of cells or any expected count < 1), two-sided Fisher’s exact test was applied.

Effect sizes were reported as odds ratios (OR) with 95% confidence intervals (CI). Given the case–control design, ORs were considered the primary measure of associa-tion, while risk ratios (RR) derived from sample proportions were provided for refer-ence only.

## 5. Conclusions

Based on the analysis of *HLA-DQB1* and *HLA-DRB1* allele associations with suicidal behavior in a cohort of 196 Transylvanian subjects (98 cases and 98 controls), our exploratory study suggests several potential genetic associations. We identified several genotypes and haplotypes potentially associated with increased susceptibility to suicidal behavior: the HLA-DQB1*02/*06 (RR 1.60) and the HLA-DRB1*11/*15 (RR 1.60) genotypes; the HLA-DRB1*15~DQB1*03 haplotype (RR 1.58). Conversely, some alleles, genotypes, and haplotypes potentially exerting a protective influence were also observed: the HLA-DRB1*04 allele (RR 0.61); the HLA-DQB1*02/*05 (RR 0.61) and HLA-DRB1*01/*03 (RR 0.33) genotypes; the HLA-DRB1*04~DQB1*03 haplotype (RR 0.62).

To validate these exploratory findings and establish a more comprehensive understanding, further large-scale research is warranted. These subsequent studies should aim to develop a comprehensive database on HLA allele frequency across diverse ethnic subgroups and all regions of the Romanian population. The identification and validation of new biological markers influencing suicidality, such as the ones suggested here, could be instrumental in implementing effective prevention strategies and advancing therapeutic schemes.

## Figures and Tables

**Table 1:** Odds ratio (OR) and relative risk (RR) of being associated with suicidal behaviors for the *HLA-DQB1* alleles.

No.	*DQB1* Allele	RR	CI95%	OR	CI95%	*p*
1	DQB1*02	0.97	0.74–1.26	0.93	0.56–1.56	0.90
2	DQB1*03	0.96	0.78–1.18	0.92	0.61–1.38	0.75
3	DQB1*04	0.66	0.21–2.07	0.49	0.09–2.73	0.68
4	DQB1*05	1.04	0.84–1.29	1.08	0.69–1.68	0.82
5	DQB1*06	1.09	0.85–1.40	1.20	0.71–2.03	0.59

**Table 2:** Odds ratio (OR) and relative risk (RR) of being associated with suicidal behaviors for the *HLA-DQB1* genotypes.

No.	*DQB1* Genotype	RR	CI95%	OR	CI95%	*p*
1	DQB1*02/*02	1.34	0.75–2.38	2.02	0.37–11.16	0.68
2	DQB1*02/*03	0.93	0.70–1.23	0.86	0.50–1.47	0.68
3	DQB1*02/*04	2.01	1.82–2.22	-	Undefined	0.48
4	DQB1*02/*05	0.61	0.39–0.95	0.43	0.22–0.83	0.02 *
5	DQB1*02/*06	1.60	1.22–2.09	3.69	1.19–11.42	0.03 *
6	DQB1*03/*03	0.91	0.65–1.25	0.83	0.45–1.52	0.64
7	DQB1*03/*04	-	Undefined	-	Undefined	0.48
8	DQB1*03/*05	1.14	0.90–1.44	1.31	0.79–2.20	0.36
9	DQB1*03/*06	0.95	0.70–1.30	0.91	0.50–1.65	0.88
10	DQB1*04/*05	0.66	0.21–2.07	0.49	0.09–2.73	0.68
11	DQB1*04/*06	-	Undefined	-	Undefined	0.48
12	DQB1*05/*05	1.37	1.04–1.80	2.11	0.96–4.64	0.09
13	DQB1*05/*06	0.87	0.58–1.30	0.76	0.37–1.58	0.58
14	DQB1*06/*06	1.26	0.85–1.87	1.70	0.61–4.78	0.44

* Statistical significance for *p* < 0.05.

**Table 3:** Odds ratio (OR) and relative risk (RR) of being associated with suicidal behaviors observed for the *HLA-DRB1* alleles.

No.	*DRB1* Allele	RR	CI95%	OR	CI95%	*p*
1	DRB1*01	1.06	0.77–1.44	1.12	0.58–2.15	0.87
2	DRB1*03	0.85	0.57–1.26	0.73	0.36–1.47	0.48
3	DRB1*04	0.61	0.37–1.00	0.43	0.20–0.90	0.03 *
4	DRB1*07	1.16	0.88–1.54	1.38	0.72–2.63	0.41
5	DRB1*08	0.66	0.21–2.07	0.49	0.09–2.73	0.68
6	DRB1*09	-	Undefined	-	Undefined	1
7	DRB1*10	1	0.37–2.68	1	0.14–7.17	1
8	DRB1*11	0.94	0.73–1.21	0.88	0.54–1.44	0.71
9	DRB1*12	1.68	1.16–2.44	5.10	0.59–44.10	0.22
10	DRB1*13	0.97	0.69–1.36	0.94	0.49–1.83	1
11	DRB1*14	0.94	0.56–1.57	0.88	0.33–2.34	1
12	DRB1*15	1.23	0.95–1.60	1.58	0.84–2.97	0.20
13	DRB1*16	1.16	0.88–1.54	1.38	0.72–2.63	0.41

* Statistical significance for *p* < 0.05.

**Table 4:** Correlations with psychosocial determinants identified in the case group for the *HLA-DQB1* alleles.

Determinants	Allele Indicator	DQB1*02	DQB1*03	DQB1*04	DQB1*05	DQB1*06
Social/familial problems	Frequency	44.4%	50%	100%	41.7%	57.7%
	Pearson X^2^	0.228	0.064	1.066	0.925	1.063
	2-tailed *p*	0.633	0.801	0.302 ^b,c^	0.336	0.302
Psychiatric pathology	Frequency	85.2%	72.0%	100%	77.8%	92.3%
	Pearson X^2^	0.562	3.111 ^b,c^	0.252	0.150	3.023
	2-tailed *p*	0.453	0.078	0.616	0.699	0.082
Trauma	Frequency	41.2%	42.0%	0%	54.5%	47.2%
	Pearson X^2^	0.372	0.649	1.716	2.291	0.030
	2-tailed *p*	0.542	0.421	0.190 ^b,c^	0.130	0.862
Abuse	Frequency	35.3%	26.1%	0%	41.8%	41.7%
	Pearson X^2^	0.007	3.482	1.073	1.713	0.946
	2-tailed *p*	0.936	0.062	0.300 ^b,c^	0.191	0.331
Chronic diseases	Frequency	67.6%	63.8%	50%	61.8%	77.8%
	Pearson X^2^	0.032	0.312	0.241	0.696	2.589
	2-tailed *p*	0.858	0.576	0.623 ^b,c^	0.404	0.108
Cancer	Frequency	0%	2.9%	0%	0%	5.6%
	Pearson X^2^	0.857	0.392	0.042	1.593	2.725
	2-tailed *p*	0.355 ^b,c^	0.531 ^b^	0.837 ^b,c^	0.207 ^b^	0.099 ^b,c^
Death of close relatives	Frequency	70.6%	52.2%	100%	49.1%	52.8%
	Pearson X^2^	3.988	0.369	1.646	1.117	0.096
	2-tailed *p*	0.046 *	0.544	0.199 ^b,c^	0.291	0.756
Disillusionment	Frequency	67.6%	55.1%	100%	60%	61.1%
	Pearson X^2^	0.951	1.170	1.336	0.001	0.015
	2-tailed *p*	0.329	0.279	0.248 ^b,c^	0.971	0.902
Depression	Frequency	94.1%	88.4%	100%	90.9%	97.2%
	Pearson X^2^	0.285	1.672	0.180	0.088	1.706
	2-tailed *p*	0.593 ^b^	0.196	0.672 ^b,c^	0.767 ^b^	0.191 ^b^
Self-harm	Frequency	52.9%	39.1%	0%	40%	52.8%
	Pearson X^2^	1.372	0.975	1.580	0.467	1.419
	2-tailed *p*	0.241	0.324	0.209 ^b,c^	0.494	0.234
Alcohol abuse	Frequency	47.1%	40.6%	50%	40%	52.8%
	Pearson X^2^	0.169	0.470	0.031	0.467	1.419
	2-tailed *p*	0.681	0.493	0.861 ^b,c^	0.494	0.234
Drug addiction	Frequency	38.2%	23.2%	0%	38.2%	38.9%
	Pearson X^2^	0.583	4.338	0.980	1.063	0.780
	2-tailed *p*	0.445	0.037 *	0.322 ^b,c^	0.303	0.377
Compulsive eating	Frequency	17.6%	14.5%	0%	14.5%	5.6%
	Pearson X^2^	0.686	0.139	0.309	0.109	2.278
	2-tailed *p*	0.407 ^b^	0.709	0.578 ^b,c^	0.741	0.131 ^b^
Lack of appetite	Frequency	70.6%	72.5%	100%	70.9%	69.4%
	Pearson X^2^	0.014	0.056	0.808	0.010	0.085
	2-tailed *p*	0.905	0.813	0.369 ^b,c^	0.920	0.771
Obsessive ideas	Frequency	73.5%	78.3%	100%	83.6%	69.4%
	Pearson X^2^	0.382	0.031	0.585	1.626	1.665
	2-tailed *p*	0.536	0.861	0.444 ^b,c^	0.202	0.197
Memory dysfunction	Frequency	76.5%	62.3%	100%	60%	83.3%
	Pearson X^2^	1.249	1.801	0.935	2.475	4.567
	2-tailed *p*	0.264	0.180	0.334 ^b,c^	0.116	0.033 *

* The correlation is significant below the 0.05 threshold. ^b^. More than 20% of the values present expected frequencies below 5. The chi-square may be invalidated. ^c^. The minimum expected frequency is less than 1. The chi-square may be invalidated.

**Table 5:** Correlations with psychosocial determinants identified in the case group for the HLA-DRB1*01–HLA-DRB1*10 alleles.

Determinants	Allele Indicator	*DRB1* *01	*DRB1* *03	*DRB1* *04	*DRB1* *07	*DRB1* *08	*DRB1* *10
Social/familial problems	Frequency	23.5%	30%	50%	52.6%	100%	0%
	Pearson X^2^	4.858	1.487	0.009	0.145	1.066	
	2-tailed *p*	0.028 *	0.223 ^b^	0.925 ^b^	0.703	0.302 ^b,c^	
Psychiatric pathology	Frequency	76.5%	70%	60%	94.7%	100%	0%
	Pearson X^2^	0.151	0.673	2.692	2.984	0.252	
	2-tailed *p*	0.698 ^b^	0.412 ^b^	0.101 ^b^	0.084 ^b^	0.616 ^b,c^	
Trauma	Frequency	52.4%	33.3%	36.4%	41.7%	0%	50%
	Pearson X^2^	0.396	1.036	0.428	0.199	1.716	0.014
	2-tailed *p*	0.529	0.309	0.513	0.655	0.190 ^b,c^	0.907 ^b,c^
Abuse	Frequency	33.3%	26.7%	36.4%	37.5%	0%	0%
	Pearson X^2^	0.019	0.462	0.014	0.095	1.073	1.073
	2-tailed *p*	0.890	0.497	0.905 ^b^	0.758	0.300 ^b,c^	0.300 ^b,c^
Chronic diseases	Frequency	61.9%	73.3%	54.5%	62.5%	50%	100%
	Pearson X^2^	0.206	0.357	0.724	0.179	0.241	1.026
	2-tailed *p*	0.650	0.550	0.395 ^b^	0.672	0.623 ^b,c^	0.311 ^b,c^
Cancer	Frequency	0%	0%	0%	0%	0%	0%
	Pearson X^2^	0.490	0.338	0.243	0.570	0.042	0.042
	2-tailed *p*	0.484 ^b,c^	0.561 ^b,c^	0.622 ^b,c^	0.450 ^b,c^	0.837 ^b,c^	0.837 ^b,c^
Death of close relatives	Frequency	52.4%	53.3%	63.6%	70.8%	100%	50%
	Pearson X^2^	0.070	0.021	0.343	2.736	1.646	0.021
	2-tailed *p*	0.791	0.886	0.558 ^b^	0.098	0.199 ^b,c^	0.884 ^b,c^
Disillusionment	Frequency	66.7%	40%	36.4%	79.2%	100%	0%
	Pearson X^2^	0.410	2.767	2.765	4.105	1.336	3.057
	2-tailed *p*	0.522	0.096	0.096 ^b^	0.043 *	0.248 ^b,c^	0.080 ^b,c^
Depression	Frequency	100%	73.3%	90.9%	100%	100%	50%
	Pearson X^2^	2.091	7.418	0.013	2.431	0.180	4.718
	2-tailed *p*	0.148 ^b^	0.006 *^,b^	0.908 ^b,c^	0.119 ^b^	0.672 ^b,c^	0.030 *^,b,c^
Self-harm	Frequency	47.6%	33.3%	36.4%	54.2%	0%	0%
	Pearson X^2^	0.134	0.733	0.267	1.176	1.580	1.580
	2-tailed *p*	0.715	0.392	0.605 ^b^	0.278	0.209 ^b,c^	0.209 ^b,c^
Alcohol abuse	Frequency	42.9%	53.3%	45.5%	41.7%	50%	0%
	Pearson X^2^	0.010	0.590	0.012	0.054	0.031	1.580
	2-tailed *p*	0.921	0.443	0.914 ^b^	0.816	0.861 ^b,c^	0.209 ^b,c^
Drug addiction	Frequency	47.6%	26.7%	27.3%	45.8%	0%	0%
	Pearson X^2^	2.396	0.265	0.153	2.160	0.980	0.980
	2-tailed *p*	0.122	0.607 ^b^	0.695 ^b^	0.142	0.322 ^b,c^	0.322 ^b,c^
Compulsive eating	Frequency	4.8%	13.3%	27.3%	20.8%	0%	0%
	Pearson X^2^	1.478	0	1.987	1.361	0.309	0.309
	2-tailed *p*	0.224 ^b^	0.994 ^b^	0.159 ^b^	0.243 ^b^	0.578 ^b,c^	0.578 ^b,c^
Lack of appetite	Frequency	81.0%	73.3%	72.7%	66.7%	100%	50%
	Pearson X^2^	1.045	0.029	0.010	0.304	0.808	0.455
	2-tailed *p*	0.307	0.865 ^b^	0.922 ^b^	0.581	0.369 ^b,c^	0.500 ^b,c^
Obsessive ideas	Frequency	90.5%	53.3%	81.8%	79.2%	100%	50%
	Pearson X^2^	2.257	5.472	0.122	0.041	0.585	0.881
	2-tailed *p*	0.133 ^b^	0.019 *^,b^	0.727 ^b^	0.840	0.444 ^b,c^	0.348 ^b,c^
Memory dysfunction	Frequency	71.4%	66.7%	45.5%	79.2%	100%	50%
	Pearson X^2^	0.102	0.022	2.829	1.475	0.935	0.315
	2-tailed *p*	0.750	0.883 ^b^	0.093 ^b^	0.225	0.334 ^b,c^	0.575 ^b,c^

* The correlation is significant below the 0.05 threshold. ^b^. More than 20% of the values present expected frequencies below 5. The chi-square may be invalidated. ^c^. The minimum expected frequency is less than 1. The chi-square may be invalidated.

**Table 6:** Correlations with psychosocial determinants identified in the case group for the HLA-DRB1*11–HLA-DRB1*16 alleles.

Determinants	Allele Indicator	*DRB1* *11	*DRB1* *12	*DRB1* *13	*DRB1* *14	*DRB1* *15	*DRB1* *16
Social/familial problems	Frequency	48.3%	100%	50%	33.3%	60.9%	58.3%
	Pearson X^2^	0.001	3.246	0.009	0.583	1.666	0.501
	2-tailed *p*	0.971	0.072 ^b^	0.925 ^b^	0.445 ^b^	0.197	0.479
Psychiatric pathology	Frequency	72.4%	66.7%	90%	100%	95.7%	58.3%
	Pearson X^2^	1.316	0.341	0.673	1.567	4.214	3.851
	2-tailed *p*	0.251	0.559 ^b,c^	0.412 ^b^	0.211 ^b^	0.04 *^,b^	0.05 *^,b^
Trauma	Frequency	39.5%	60%	52.6%	62.5%	48.1%	54.2%
	Pearson X^2^	0.788	0.410	0.382	0.923	0.063	0.749
	2-tailed *p*	0.375	0.522 ^b^	0.537	0.337 ^b^	0.802	0.387
Abuse	Frequency	18.4%	40%	36.8%	62.5%	44.4%	45.8%
	Pearson X^2^	5.509	0.064	0.043	2.846	1.314	1.498
	2-tailed *p*	0.019 *	0.801 ^b^	0.836	0.092 ^b^	0.252	0.221
Chronic diseases	Frequency	65.8%	60%	78.9%	50%	74.1%	62.5%
	Pearson X^2^	0.006	0.092	1.501	0.995	0.842	0.179
	2-tailed *p*	0.938	0.762 ^b^	0.221	0.318 ^b^	0.359	0.672
Cancer	Frequency	2.6%	20%	5.3%	0%	3.7%	0%
	Pearson X^2^	0.082	8.278	1.093	0.174	0.433	0.570
	2-tailed *p*	0.774 ^b,c^	0.004 *^,b,c^	0.296 ^b,c^	0.677 ^b,c^	0.510 ^b,c^	0.450 ^b,c^
Death of close relatives	Frequency	50%	40%	52.6%	75.0%	55.6%	41.7%
	Pearson X^2^	0.496	0.473	0.052	1.335	0.003	1.995
	2-tailed *p*	0.481	0.492 ^b^	0.820	0.248 ^b^	0.959	0.158
Disillusionment	Frequency	52.6%	60%	47.4%	75.0%	85.2%	50%
	Pearson X^2^	1.128	0	1.447	0.762	8.156	1.189
	2-tailed *p*	0.288	0.992 ^b^	0229	0.383 ^b^	0.004 *	0.276
Depression	Frequency	84.2%	80%	100%	100%	96.3%	91.7%
	Pearson X^2^	3.657	0.959	1.870	0.741	0.831	0.001
	2-tailed *p*	0.056 ^b^	0.327 ^b,c^	0.171 ^b^	0.389 ^b,c^	0.362 ^b^	0.974 ^b^
Self-harm	Frequency	44.7%	0%	47.4%	37.5%	66.7%	29.2%
	Pearson X^2^	0.014	4.011	0.104	0.138	6.604	2.403
	2-tailed *p*	0.905	0.045 *^,b^	0.747	0.711 ^b^	0.010 *	0.121
Alcohol abuse	Frequency	36.8%	40%	52.6%	37.5%	44.4%	50%
	Pearson X^2^	0.948	0.031	0.655	0.138	0.004	0.416
	2-tailed *p*	0.330	0.860 ^b^	0.418	0.711 ^b^	0.949	0.519
Drug addiction	Frequency	23.7%	20%	26.3%	37.5%	44.4%	25.0%
	Pearson X^2^	1.724	0.374	0.384	0.089	1.980	0.728
	2-tailed *p*	0.189	0.541 ^b^	0.535	0.765 ^b^	0.159	0.393
Compulsive eating	Frequency	7.9%	0%	5.3%	37.5%	7.4%	25.0%
	Pearson X^2^	1.182	0.785	1.171	4.257	0.934	3.273
	2-tailed *p*	0.277	0.376 ^b,c^	0.279 ^b^	0.039 *^,b^	0.334 ^b^	0.070 ^b^
Lack of appetite	Frequency	78.9%	20%	68.4%	62.5%	74.1%	66.7%
	Pearson X^2^	1.306	6.650	0.093	0.326	0.107	0.304
	2-tailed *p*	0.253	0.010 *^,b^	0.760	0.568 ^b^	0.743	0.581
Obsessive ideas	Frequency	73.7%	80%	63.2%	100%	85.2%	79.2%
	Pearson X^2^	0.405	0.018	2.504	2.414	1.048	0.041
	2-tailed *p*	0.525	0.894 ^b^	0.114 ^b^	0.120 ^b^	0.306	0.840
Memory dysfunction	Frequency	57.9%	80%	78.9%	62.5%	81.5%	58.3%
	Pearson X^2^	2.391	0.321	1.089	0.133	2.490	1.273
	2-tailed *p*	0.122	0.571 ^b^	0.297	0.716 ^b^	0.115	0.259

* The correlation is significant below the 0.05 threshold. ^b^. More than 20% of the values present expected frequencies below 5. The chi-square may be invalidated. ^c^. The minimum expected frequency is less than 1. The chi-square may be invalidated.

## Data Availability

The data generated and analyzed in this study are presented in the article. Supplementary inquiries may be addressed to the corresponding author.

## Supplementary Materials

The following supporting information can be downloaded at: https://www.mdpi.com/article/10.3390/ijms262010181/s1.
