# Supplementary material for: HLA Class II Alleles and Suicidal Behavior: Evidence from a Case–Control Study
Source: Int J Mol Sci. 2025 Oct 20;26(20):10181. doi: 10.3390/ijms262010181 (PMC12563147; doi:10.3390/ijms262010181)
Supplement: Supplementary file 1 [file ijms-26-10181-s001.zip › ijms-3906609-supplementary.pdf]

Table S1. Odds ratio (OR) and relative risk (RR) of being associated with suicidal behaviors observed for the *HLA-DRB1* genotypes

No.	*DRB1* genotype	RR	CI95%	OR	CI95%	*p*
1	*DRB1*01/*01*	2.02	1.83-2.23	-	Undefined	0.13
2	***DRB1*01/*03***	**0.33**	**0.09-1.16**	**0.19**	**0.04-0.89**	**0.04***
3	*DRB1*01/*04*	1	0.37-2.68	1	0.14-7.17	1
4	*DRB1*01/*07*	0.80	0.37-1.71	0.66	0.18-2.38	0.75
5	*DRB1*01/*08*	1	0.37-2.68	1	0.14-7.17	1
6	*DRB1*01/*11*	1	0.61-1.65	1	0.37-2.72	1
7	*DRB1*01/*13*	1	0.50-2.01	1	0.25-4.06	1
8	*DRB1*01/*14*	-	Undefined	-	Undefined	0.48
9	*DRB1*01/*15*	1.52	1.00-2.29	3.06	0.61-15.37	0.28
10	*DRB1*01/*16*	1.52	1.00-2.29	3.06	0.61-15.37	0.28
11	*DRB1*03/*03*	2.02	1.83-2.23	-	Undefined	0.13
12	*DRB1*03/*04*	1	0.56-1.78	1	0.32-3.16	1
13	*DRB1*03/*07*	0.66	0.21-2.07	0.49	0.09-2.73	0.68
14	*DRB1*03/*11*	0.66	0.34-1.27	0.48	0.18-1.32	0.23
15	*DRB1*03/*12*	2.01	1.82-2.22	-	Undefined	0.48
16	*DRB1*03/*13*	-	Undefined	-	Undefined	0.48
17	*DRB1*03/*14*	2.01	1.82-2.22	-	Undefined	0.48
18	*DRB1*03/*15*	1	0.37-2.68	1	0.14-7.17	1
19	*DRB1*03/*16*	-	Undefined	-	Undefined	0.13
20	*DRB1*04/*04*	-	Undefined	-	Undefined	0.13
21	*DRB1*04/*07*	1	0.56-1.78	1	0.32-3.16	1
22	*DRB1*04/*11*	0.56	0.24-1.30	0.39	0.12-1.26	0.17
23	*DRB1*04/*13*	-	Undefined	-	Undefined	0.01
24	*DRB1*04/*14*	-	Undefined	-	Undefined	0.48
25	*DRB1*04/*15*	0.66	0.21-2.07	0.49	0.09-2.73	0.68
26	*DRB1*04/*16*	1	0.37-2.68	1	0.14-7.17	1
27	*DRB1*07/*07*	1	0.37-2.68	1	0.14-7.17	1
28	*DRB1*07/*08*	2.01	1.82-2.22	-	Undefined	0.48
29	*DRB1*07/*11*	1.45	1.03-2.05	2.58	0.80-8.37	0.17
30	*DRB1*07/*13*	2.05	1.85-2.28	-	Undefined	0.00
31	*DRB1*07/*14*	-	Undefined	-	Undefined	0.48
32	*DRB1*07/*15*	1.52	1.00-2.29	3.06	0.61-15.37	0.28
33	*DRB1*07/*16*	0.66	0.29-1.48	0.49	0.14-1.65	0.38
34	*DRB1*08/*11*	-	Undefined	-	Undefined	0.48
35	*DRB1*08/*13*	-	Undefined	-	Undefined	0.48
36	*DRB1*08/*15*	-	Undefined	-	Undefined	0.48
37	*DRB1*09/*16*	-	Undefined	-	Undefined	0.48
38	*DRB1*10/*11*	2.01	1.82-2.22	-	Undefined	0.48
39	*DRB1*10/*13*	-	Undefined	-	Undefined	0.13
40	*DRB1*10/*16*	2.01	1.82-2.22	-	Undefined	0.48
41	*DRB1*11/*11*	0.90	0.57-1.45	0.82	0.35-1.96	0.83

42	*DRB1*11/*13*	0.66	0.34-1.27	0.48	0.18-1.32	0.23
43	*DRB1*11/*14*	0.66	0.21-2.07	0.49	0.09-2.73	0.68
**44**	***DRB1*11/*15***	**1.70**	**1.30-2.24**	**5.22**	**1.13-24.12**	**0.04***
45	*DRB1*11/*16*	1.15	0.72-1.83	1.35	0.46-3.96	0.79
46	*DRB1*12/*15*	2.02	1.83-2.23	-	Undefined	0.13
47	*DRB1*12/*16*	1.34	0.75-2.38	2.02	0.37-11.16	0.68
48	*DRB1*13/*13*	2.03	1.84-2.25	-	Undefined	0.04
49	*DRB1*13/*14*	2.01	1.82-2.22	-	Undefined	0.48
50	*DRB1*13/*15*	0.49	0.15-1.65	0.33	0.07-1.64	0.28
51	*DRB1*13/*16*	1	0.37-2.68	1	0.14-7.17	1
52	*DRB1*14/*15*	1	0.56-1.78	1	0.32-3.16	1
53	*DRB1*14/*16*	1.34	0.75-2.38	2.02	0.37-11.16	0.68
54	*DRB1*15/*15*	1.63	1.17-2.25	4.13	0.87-19.69	0.11
55	*DRB1*15/*16*	-	Undefined	-	Undefined	0.04
56	*DRB1*16/*16*	2.04	1.84-2.26	-	Undefined	0.01

*Statistical significance for $p<0.05$.

Table S2. Odds ratio (OR) and relative risk (RR) of being associated with suicidal behaviors observed for the *HLA-DRB1~DQB1* haplotypes

No.	*DRB1~DQB1* haplotypes	RR	CI95%	OR	CI95%	*p*
1	*DRB1*01~DQB1*02*	0.54	0.21-1.43	0.37	0.10-1.41	0.22
2	*DRB1*01~DQB1*03*	1.08	0.65-1.79	1.17	0.39-3.51	1
3	*DRB1*01~DQB1*04*	1	0.25-4.01	1	0.06-16.04	1
4	*DRB1*01~DQB1*05*	1.13	0.87-1.47	1.31	0.73-2.35	0.46
5	*DRB1*01~DQB1*06*	1.34	0.76-2.36	2.01	0.37-11.04	0.68
6	*DRB1*03~DQB1*02*	0.83	0.56-1.22	0.70	0.36-1.38	0.39
7	*DRB1*03~DQB1*03*	1	0.64-1.56	1	0.41-2.43	1
8	*DRB1*03~DQB1*05*	0.72	0.33-1.59	0.57	0.16-1.95	0.54
9	*DRB1*03~DQB1*06*	0.67	0.13-3.30	0.50	0.05-5.52	1
10	*DRB1*04~DQB1*02*	0.71	0.35-1.44	0.55	0.18-1.66	0.42
11	***DRB1*04~DQB1*03***	**0.62**	**0.40-0.97**	**0.45**	**0.23-0.86**	**0.02***
12	*DRB1*04~DQB1*05*	0.80	0.27-2.34	0.66	0.11-4.00	1
13	*DRB1*04~DQB1*06*	0.28	0.05-1.74	0.16	0.02-1.37	0.13
14	*DRB1*07~DQB1*02*	1.20	0.93-1.56	1.50	0.80-2.83	0.27
15	*DRB1*07~DQB1*03*	1.05	0.69-1.59	1.10	0.46-2.63	1
16	*DRB1*07~DQB1*04*	2.00	1.87-2.15	-	Undefined	1
17	*DRB1*07~DQB1*05*	0.77	0.38-1.53	0.62	0.20-1.91	0.58
18	*DRB1*07~DQB1*06*	1.73	1.26-2.35	6.08	0.73-50.72	0.13
19	*DRB1*08~DQB1*02*	2.00	1.87-2.15	-	Undefined	1
20	*DRB1*08~DQB1*03*	-	Undefined	-	Undefined	1
21	*DRB1*08~DQB1*04*	0.66	0.21-2.07	0.50	0.09-2.73	0.68
22	*DRB1*08~DQB1*05*	0.67	0.13-3.30	0.50	0.05-5.52	1
23	*DRB1*08~DQB1*06*	-	Undefined	-	Undefined	1
24	*DRB1*09~DQB1*03*	-	Undefined	-	Undefined	1
25	*DRB1*09~DQB1*05*	-	Undefined	-	Undefined	1
26	*DRB1*10~DQB1*03*	1	0.25-4.01	1	0.06-16.04	1
27	*DRB1*10~DQB1*05*	1.20	0.59-2.47	1.50	0.25-9.05	1
28	*DRB1*10~DQB1*06*	-	Undefined	-	Undefined	1
29	*DRB1*11~DQB1*02*	0.93	0.54-1.61	0.87	0.31-2.43	1
30	*DRB1*11~DQB1*03*	0.92	0.74-1.13	0.85	0.57-1.26	0.47
31	*DRB1*11~DQB1*04*	-	Undefined	-	Undefined	1
32	*DRB1*11~DQB1*05*	1.10	0.74-1.65	1.23	0.50-3.00	0.82
33	*DRB1*11~DQB1*06*	1	0.57-1.77	1	0.32-3.13	1
34	*DRB1*12~DQB1*03*	1.61	1.03-2.50	4.03	0.45-36.23	0.37
35	*DRB1*12~DQB1*05*	1.61	1.03-2.50	4.03	0.45-36.23	0.37
36	*DRB1*12~DQB1*06*	2.01	1.87-2.15	-	Undefined	0.48
37	*DRB1*13~DQB1*02*	1.68	1.16-2.41	5.05	0.59-43.44	0.22
38	*DRB1*13~DQB1*03*	0.69	0.38-1.27	0.53	0.21-1.34	0.26
39	*DRB1*13~DQB1*04*	-	Undefined	-	Undefined	1
40	*DRB1*13~DQB1*05*	0.66	0.26-1.68	0.50	0.12-2.00	0.50
41	*DRB1*13~DQB1*06*	1.10	0.83-1.46	1.22	0.66-2.28	0.63
42	*DRB1*14~DQB1*02*	1	0.25-4.01	1	0.06-16.04	1

43	*DRB1*14~DQB1*03*	1.14	0.60-2.18	1.34	0.30-6.01	1
44	*DRB1*14~DQB1*05*	0.81	0.49-1.35	0.69	0.29-1.62	0.52
45	*DRB1*14~DQB1*06*	1.34	0.60-2.98	2.01	0.18-22.20	1
46	*DRB1*15~DQB1*02*	1.61	1.03-2.50	4.03	0.45-36.23	0.37
47	***DRB1*15~DQB1*03***	**1.58**	**1.22-2.04**	**3.59**	**1.17-11.01**	**0.03***
48	*DRB1*15~DQB1*04*	-	Undefined	-	Undefined	1
49	*DRB1*15~DQB1*05*	0.95	0.60-1.50	0.91	0.38-2.16	1
50	*DRB1*15~DQB1*06*	1.17	0.90-1.51	1.39	0.76-2.56	0.36
51	*DRB1*16~DQB1*02*	0.50	0.15-1.66	0.33	0.07-1.64	0.29
52	*DRB1*16~DQB1*03*	1.41	1.05-1.90	2.38	0.91-6.27	0.11
53	*DRB1*16~DQB1*05*	1.23	0.98-1.56	1.60	0.89-2.85	0.15
54	*DRB1*16~DQB1*06*	0.40	0.07-2.30	0.25	0.03-2.23	0.37

*Statistical significance for $p<0.05$.
